# Biological functionality of non-functional protein kinases

**DOI:** 10.1093/jxb/eraf059

**Published:** 2025-04-09

**Authors:** Matthieu H A J Joosten

**Affiliations:** Laboratory of Phytopathology, Wageningen University, Droevendaalsesteeg 1, 6708 PB Wageningen, The Netherlands

**Keywords:** ABA receptor kinase, CARK, CPK28, cytosolic phosphorylation, kinase-inactive, calcium-dependent protein kinase, MAZZA, receptor-like cytoplasmic kinase (RLCK)

## Abstract

This article comments on:

**Gonçalves Dias M, Dharmasena T, Gonzalez-Ferrer C, Maika JE, Miguel VN, Dou R, Rodriguez Gallo MC, Bredow M, Siegel KR, Uhrig RG, Simon R, Monaghan J.** 2025. Catalytically inactive subgroup VIII receptor-like cytoplasmic kinases regulate the immune-triggered oxidative burst in *Arabidopsis thaliana*. Journal of Experimental Botany **76**, 1553–1568. https://doi.org/10.1093/jxb/erae486

This article comments on:

**Gonçalves Dias M, Dharmasena T, Gonzalez-Ferrer C, Maika JE, Miguel VN, Dou R, Rodriguez Gallo MC, Bredow M, Siegel KR, Uhrig RG, Simon R, Monaghan J.** 2025. Catalytically inactive subgroup VIII receptor-like cytoplasmic kinases regulate the immune-triggered oxidative burst in *Arabidopsis thaliana*. Journal of Experimental Botany **76**, 1553–1568. https://doi.org/10.1093/jxb/erae486

**To provide a fast and versatile response to adverse conditions, the plant proteome is subjected to swift post-translational modifications, of which phosphorylation is the most significant. Target proteins are phosphorylated by protein kinases, and this generally results in the activation of a cascade of phosphorylation events that eventually trigger a particular output. Gonçalves Dias *et al.* (2025) studied a subgroup of so-called receptor-like cytoplasmic kinases (RLCKs) that play a role in plant immunity to microbial pathogens and in development. Strikingly, the authors showed that for their biological function *in planta* these RLCKs do not require catalytic protein kinase activity.**

In addition to completing a developmental programme with the aim to eventually reproduce, plants must cope with adverse abiotic factors, such as heat and drought, and biotic agents, such as microbial pathogens and pests. To maintain homeostasis and to provide a swift and flexible response to perturbations caused by (a)biotic factors, the plant proteome is continuously subjected to intricate post-translational modifications, of which protein phosphorylation, that can be reversible, has the largest effects (Olsen *et al.*, 2006). Target proteins are phosphorylated by enzymes referred to as kinases, and this generally results in the activation of a cascade of phosphorylation events involving a chain of signalling partners and which eventually trigger a particular output.

Receptors at the plant cell surface form the first line of defence and they can recognize extracellular immunogenic patterns derived from pathogenic microbes (van der Burgh and Joosten, 2019). One of the very first defence responses upon recognition of an immunogenic pattern is the production of toxic reactive oxygen species (ROS) that diffuse to the apoplast and thereby provide a first barrier for the pathogen to overcome. In addition, ROS are key signalling molecules in local and systemic immune responses. In the model plant *Arabidopsis thaliana* (Arabidopsis), ROS production is catalysed by the plasma membrane (PM)-localized enzyme RESPIRATORY BURST OXIDASE HOMOLOG D (RBOHD), which is phosphorylated by the RLCK referred to as BOTRYTIS-INDUCED KINASE 1 (BIK1) (Kadota *et al.*, 2014; Li *et al.*, 2014). BIK1 is a central substrate of many cell surface receptor complexes, and BIK1 is rapidly phosphorylated upon activation of the BIK1-interacting cell surface receptor complex by its matching immunogenic pattern.

Receptor activation also results in a swift calcium (Ca^2+^) influx into the cell, leading to an increased Ca^2+^ concentration in the cytoplasm, to which so-called calcium-dependent protein kinases (CDPKs) are extremely responsive. The team of Jacqueline Monaghan studies CPK28, which is a very versatile CDPK, having a function in plant growth and development, responses to abiotic stress, and immunity (Monaghan *et al.*, 2014).

CPK28 contains an N-terminal myristoylation motif and localizes at the PM. CPK28 is a negative regulator of immune signalling, as this kinase interacts with and phosphorylates BIK1, which also associates at the PM. The amount of BIK1 protein available determines the intensity of the immune response that is triggered by cell surface receptors, and the BIK1 protein is turned over continuously to secure cellular homeostasis. Interestingly, CPK28 contributes to BIK1 turnover, as CPK28 phosphorylates the E3 ubiquitin ligases PLANT U-BOX 25 (PUB25) and PUB26, thereby enhancing their ability to polyubiquitinate the key signalling kinase BIK1. Consequently, the BIK1 protein will be degraded in the proteasome (Wang *et al.*, 2018).

Earlier, the team of Monaghan revealed that the C7 Raf-like kinases MRK1, RAF26, and RAF39 are interactors of CPK28 (Gonçalves Dias *et al.*, 2024). These Raf-like kinases play a role in stomatal opening, ROS production, and resistance to bacterial pathogens. CPK28 phosphorylates RAF26 and RAF39, and these kinases show various similarities to mitogen-activated protein kinase kinase kinases (MAPKKKs). Still, although MRK1, RAF26, and RAF39 are active kinases, they do not phosphorylate the MAPKKs of Arabidopsis.

In this issue, Gonçalves Dias *et al.* (2025) describe their studies on additional RLCKs that play a role in the immune response in addition to BIK1. RLCKs form a huge family of proteins, with 149 members in Arabidopsis. The largest RLCK family in plants is formed by the RLCK-VIIs, which also includes BIK1, and for this family it has been shown that several members play a role in immunity (Rao *et al.*, 2018). However, the authors here focused on family VIII RLCKs, named MAZZA (MAZ), and its paralogues CYTOSOLIC ABA RECEPTOR KINASE 6 (CARK6) and CARK7, as these were identified as CPK28-interacting proteins that co-purified upon affinity purification of CPK28–yellow fluorescent protein (YFP) that was stably expressed in a transgenic Arabidopsis line. Well-known members of the RLCK family VIII are the PTO-INTERACTING PROTEINS 1a and 1b (Pti1 and Pti1b) of tomato, which are both active kinases and play a role downstream of the resistance protein Pto, which is a kinase that provides resistance to the bacterium *Pseudomonas syringae* pathovar *tomato* (*Pst*) (Zhou *et al.*, 1995).

The authors first confirmed that MAZ and CARK7 are genuine CPK28 interactors by performing a split luciferase complementation assay (SLCA) and a fluorescence lifetime imaging-based Förster resonance energy transfer (FLIM-FRET) assay, after which it was shown that both CPK28 and MAZ co-localize at the PM. Subsequent *in vitro* kinase assays revealed that CPK28 is able to phosphorylate kinase-inactive variants of both MAZ and CARK7. Interestingly, in a reciprocal experiment, wild-type MAZ and CARK7 proteins were not able to phosphorylate a kinase-inactive version of CPK28. There was also no indication that MAZ and CARK7 can be converted into active kinases as a result of phosphorylation by CPK28, leading to the conclusion that both MAZ and CARK7 are kinase-inactive RLCKs. This conclusion was further substantiated by additional *in vitro* kinase assays in which MAZ and CARK7 were tested for being able to autophosphorylate. The outcome of these tests was also negative, even when both RLCKs were produced in a strain of *Escherichia coli* expressing a phosphatase that should prevent the addition of possible inhibitory phosphate groups on the recombinant proteins.

Earlier it was shown that CARKs can form homo- and heterodimers within the VIII family of RLCKs (Li *et al.*, 2022). Such dimerization might possibly determine the kinase activity of these RLCKs, and MAZ and CARK7 were therefore tested together in kinase assays, but again no catalytic activity was observed.

Both MAZ and CARK7 contain a relatively long (100 amino acids for MAZ) intrinsically disordered N-terminal domain. Possibly, this long N-terminal extension auto-inhibits the kinase activity of the protein, and therefore a deletion mutant of MAZ was tested, lacking 90 amino acids of this N-terminal extension. However, this deletion mutant also did not show any kinase activity. Most essential features for active kinases are present in MAZ and CARK7, including the activation loop, which ends with the conserved ‘APE’ motif (Nolen *et al.*, 2004). However, the activation loop starts with ‘DFN’ instead of ‘DFG’, which can be detrimental for the kinase activity of both MAZ and CARK7. Therefore, the authors generated a mutant of MAZ containing the highly conserved ‘DFG’ motif, but for that variant too no kinase activity could be detected.

Although it was eventually unambiguously shown that the MAZ and CARK proteins are kinase inactive, these RLCKs were tested for possible involvement in the immune response. For this, Arabidopsis T-DNA insertion lines were obtained for *MAZ* and *CARK6*, *CARK7*, and *CARK9*. Furthermore, *maz*/*cark6* and *cark7*/*cark9* double knockout mutants were obtained. Upon challenge with the flagellin 22 (flg22) peptide, which is derived from bacterial flagella and is perceived by the receptor-like kinase (RLK) FLAGELLIN-SENSING 2 (FLS2) present at the cell surface, all Arabidopsis mutants generated a typical ROS burst that was highly similar to that of the Col-0 wild type, except for the *maz*/*cark6* double mutant for which an increased ROS burst was observed. In all cases, there was normal MAPK activation, suggesting that this phosphorylation event occurs independently of the family VIII RLCKs. Additional tests involving the *maz*/*cark6* and *cark7*/*cark9* double knockout mutants revealed a sensitivity to the flg22 peptide of seedlings similar to Col-0, whereas the mutants also did not show an altered susceptibility to *Pst*.

Family VIII RLCKs are also involved in plant development, and it was observed earlier that a *maz* single mutant is partially resistant to CLAVATA3 (CLV3)-triggered root meristem differentiation (Blümke *et al.*, 2021). This partial insensitivity can be restored by complementation with recombinant genes expressing the MAZ protein and, strikingly, complementation with a mutant *MAZ* gene, constitutively expressing a kinase-inactive form of MAZ, also fully restored the sensitivity of the transformant for CLV3.

In conclusion, the authors show that MAZ and CARK6, which do not show any catalytic protein kinase activity *in vitro*, act as negative regulators of the immune-triggered oxidative burst (Fig. 1). Furthermore, a kinase-inactive mutant of MAZ is able to complement a *maz* knockout. These data indicate that these family VIII RLCKs do not require catalytic activity for their biological function *in planta* and that some kind of non-catalytic mechanism is involved. A similar observation was made for the ELONGATION FACTOR TU RECEPTOR (EFR), which is a kinase-active RLK that does not require kinase activity for its biological function (Mühlenbeck *et al.*, 2024). Another example is BAK1-INTERACTING RECEPTOR-LIKE KINASE 2 (BIR2), which negatively regulates BAK1-mediated immunity but lacks the essential residues and motifs to render it an active kinase. Still, the pseudokinase BIR2 is phosphorylated by BAK1 and thereby BIR2 possibly exerts it function as a negative regulator (Blaum *et al.*, 2014).

**Fig. 1.**
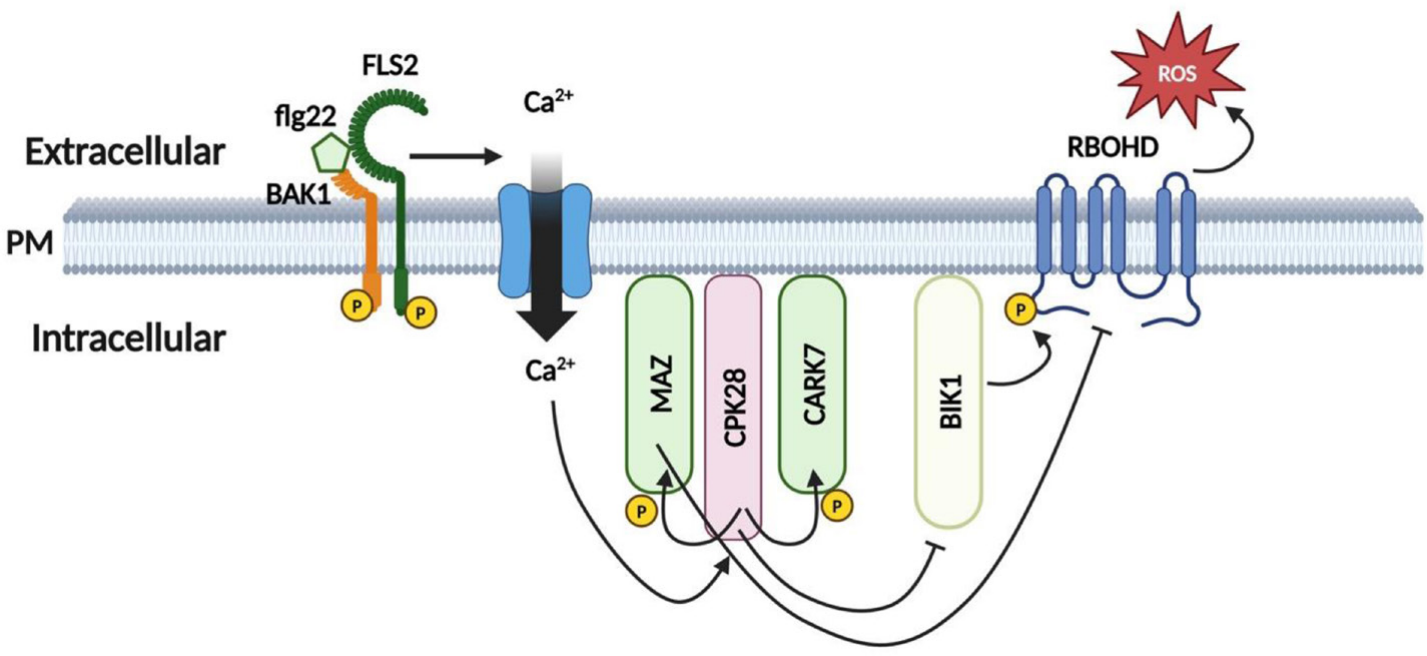
MAZZA RLCKs are negative regulators of the oxidative burst in Arabidopsis. MAZZA (MAZ) and its paralogues CARK6 and CARK7 are plasma membrane (PM)-associated RLCKs that associate with the calcium-dependent protein kinase CPK28 and lack kinase activity themselves. Upon perception of the flg22 peptide by the cell surface receptor FLS2, BAK1 is recruited and the cytoplasmic kinase domains of FLS2 and BAK1 become reciprocally phosphorylated (P in yellow circle). This results in a Ca^2+^ influx that may regulate CPK28 and subsequently its substrates MAZ and CARK7. BIK1 is an RLCK that promotes the oxidative burst (ROS) by phosphorylating the RBOHD enzyme. CPK28 inhibits BIK1 by promoting its degradation, whereas MAZ and CARK phosphorylation might cause these RLCKs to become scaffolds that can regulate the activity of the signalling complex. These kinase-inactive family VIII RLCKs might also compete with kinase-active RLCKs for recruitment to crucial signalling partners, thereby possibly negatively regulating the ROS burst. See text for further details. Picture created in https://help.biorender.com/hc/en-gb/articles/17605511350685-How-to-cite-your-BioRender-figure.

Similarly, phosphorylation of MAZ and CARK7 by CPK28 might result in a stable conformation of these RLCKs that thereby become scaffolds that can allosterically regulate the biological activity of interacting signalling partners or promote the formation of an active signalling complex. Alternatively, these kinase-inactive family VIII RLCKs may compete with active RLCKs for interacting with crucial signalling partners, thereby negatively regulating, for example, the immune-triggered ROS burst.

Step by step, we are learning more about the fascinating world of the RLCKs that play a role in plant development and immunity. However, many questions remain. Why are there so many different RLCKs? How is their biological function regulated? What is the evolutionary advantage of such a plethora of cytoplasmic kinases? Hopefully, we will soon be able to employ artificial intelligence in pathway prediction, eventually allowing us to decipher the complete immune signalling pathway of plants.
